# Nonlinear transcriptomic responses to compounded environmental changes across temperature and resources in a pest beetle, *Callosobruchus maculatus* (Coleoptera: Chrysomelidae)

**DOI:** 10.1093/jisesa/ieae106

**Published:** 2024-12-13

**Authors:** Beth A McCaw, Aoife M Leonard, Lesley T Lancaster

**Affiliations:** School of Biological Sciences, University of Aberdeen, Aberdeen, UK; Department of Ecoscience, Aarhus University, 4000 Roskilde, Denmark; School of Biological Sciences, University of Aberdeen, Aberdeen, UK

**Keywords:** environmental change, insect, life history, multidimensional, RNA-Sequencing

## Abstract

Many species are experiencing drastic and multidimensional changes to their environment due to anthropogenic events. These multidimensional changes may act nonadditively on physiological and life history responses, and thus may not be predicted by responses to single dimensional environmental changes. Therefore, work is needed to understand species’ responses to multiple aspects of change. We used whole-transcriptomic RNA-Sequencing and life history assays to uncover responses to singly-applied shifts in resource or temperature environmental dimensions, in comparison to combined, multidimensional change, in the crop pest seed beetle, *Callosobruchus maculatus*. We found that multidimensional change caused larger fecundity, developmental period and offspring viability life history changes than predicted by additive effects of 1-dimensional changes. In addition, there was little overlap between genes differentially expressed under multidimensional treatment versus under altered resource or temperature conditions alone. Moreover, 115 genes exhibited significant resource × temperature interaction effects on expression, including those involved in energy metabolism, detoxification, and enhanced formation of cuticle structural components. We conclude that single dimensional changes alone cannot determine life history and transcriptomic responses to multidimensional environmental change. These results highlight the importance of studying multidimensional environmental change for understanding the molecular and phenotypic responses that may allow organisms including insects to rapidly adapt simultaneously to multiple aspects of environmental change.

## Introduction

Many species around the world are currently experiencing environmental change which may involve alterations in the physical environmental dimension consisting of abiotic factors (for example global climate changes) and/or changes in the biotic dimension (for example resource availability through land use change). These changes in either dimension rarely change in isolation, with many circumstances leading organisms to experience simultaneous changes in multiple aspects of the environment, or multidimensional environmental change (Gotcha et al. 2018, Padda and Stahlschmidt 2022). For instance, a population expanding or shifting its range in response to temperature change may subsequently experience changes in resource availability (Pateman et al. 2012). Exposure to such drastic environmental change may induce life history changes, influencing their ability to survive and produce offspring for the next generation (Wadgymar et al. 2024). As such, it is important to understand the combined effects of multiple aspects of environmental change on physiology and life history that may enable individuals to respond to multidimensional change and survive in a heterogeneous environment (Spence and Tingley 2020, Macaulay et al. 2021, Padda and Stahlschmidt 2022).

Most studies that aim to predict insect responses to environmental change manipulate only a single dimension of the environment, for instance biotic factors like changes in resource (Desroches et al. 1997, Rêgo et al. 2020) and abiotic factors like temperature (Bale et al. 2002, Skendžić et al. 2021, Noer et al. 2023). These 2 environmental factors in particular are relevant dimensions to study in insects, to understand their distinct life history strategies for exploiting novel plants and potentially spreading their distributions, or to demonstrate the potential impacts of future climate scenarios such as increased temperature or more variable temperature regimes on ectotherm thermal tolerance, physiology and life history. Studying responses along a single dimension is therefore useful for understanding the direct effects of a given environmental change while controlling confounding variables (Padda and Stahlschmidt 2022). However, multidimensional environmental change may invoke nonadditive responses, i.e., changes to multiple dimensions can have a greater impact (synergistic) or lesser impact (antagonistic) on organismal responses compared to the impact of each single dimensional change (Folt et al. 1999, Kaunisto et al. 2016, Orr et al. 2020, 2022). Thus, studying multidimensional interactions to identify the direction of response (additive, nonadditive synergistic or nonadditive antagonistic) across species is important, because if the context dependence is not understood, predicted organismal responses may be biased by assuming some previously measured responses will occur or are transferrable across all contexts (Orr et al. 2020).

While there has been previous research on the impacts of multidimensional environmental changes in temperature and resource on phenotypic responses in insects (Stillwell et al. 2007, Diamond and Kingsolver 2010, Lee and Roh 2010, Rodgers and Gomez Isaza 2021), less information is known about how molecular responses to a single dimension of the environment are altered when one or more other dimensions are also manipulated, and whether or how potentially nonadditive molecular responses are associated with phenotypic responses to multiple aspects of environmental change. Characterizing the molecular responses to novel environments is necessary to help understand the genes involved in driving immediate phenotypic responses to the environment and thus the traits likely to be under selection (Koch and Guillaume 2020).

Insects play key roles in ecosystem functions (Halsch et al. 2021). However, insects are environmentally sensitive, and there is often variation in responses among species due to differences in phenology, life history strategies and ecological niche requirements (Lehmann et al. 2020). Therefore, while some insect species have suffered population declines due to recent environmental change (Wagner 2020), many insects are capable of rapidly adapting to environmental change, particularly agricultural insect pests (Skendžić et al. 2021). Mechanistic studies on the molecular and phenotypic responses to multidimensional environmental changes will therefore allow comparative approaches to expand our understanding of insect adaptation to environmental change.

*Callosobruchus maculatus* (F.) (Coleoptera: Chrysomelidae) is an agricultural crop pest that infests and completes its larval and pupal life cycle in stored, dried legumes (Wasserman and Futuyma 1981, Tuda et al. 2014). *Callosobruchus maculatus* is a well-established model organism previously used to study the genetic and phenotypic mechanisms underlying its abilities to rapidly colonize a wide variety of leguminous resources and thermal regimes (Gompert and Messina 2016, Sayadi et al. 2016, Messina and Gompert 2017, Price et al. 2017, Leonard and Lancaster 2020, Rêgo et al. 2020). In addition, the availability of the *C. maculatus* annotated genome (Sayadi et al. 2019) provides a critical resource for identifying specific genes involved in adaptation to novel environments. Multidimensional changes in host resource and temperature have previously been found to interactively affect body mass, growth rate and fecundity of *C. maculatus* (Stillwell et al. 2007); however, the underlying mechanisms responding to resource and temperature change was not investigated. Moreover, whole-transcriptomic RNA-Sequencing (RNA-Seq) has previously shown that differential expression of detoxifying genes may be associated with genetic trade-offs behind shifting of a single dimension (host resource) in *C. maculatus* (Messina and Gompert 2017, Rêgo et al. 2020). We aimed to study transcriptomic and life history responses during single and multidimensional environmental change, under short-term novel host resource and/or novel temperature change. We exposed individuals from the insect model system *C. maculatus* to increased temperatures and/or altered resources, and assessed changes in multiple life history traits and gene expression. Using RNA-Seq to uncover the genetic mechanisms involved in multiple environmental changes, and whether these mechanisms act multiplicatively in a multidimensional environment involving resource and temperature change, will provide a better understanding of the mechanisms behind responses to single and multidimensional environmental change in insect pests. We aimed to test whether multidimensional change has an additive or nonadditive effect on life history and gene expression in seed beetles. We predict that (i) multiple environmental changes may interactively impact gene expression, with combined changes predicted to elicit gene expression responses that do not respond to single dimensional changes, (ii) that the life history responses to single dimensional changes will be less variable than responses to simultaneous change to multiple dimensions, and (iii) these patterns may be mirrored in gene expression changes, thereby identifying key genes important for multidimensional environmental change.

## Materials and Methods

### Study System

Our stock culture of *C. maculatus* are specialist herbivores, having been originally collected from Niger and then maintained in laboratory conditions on their ancestral diet of dried cowpeas (*Vigna unguiculata*) for over 300 generations at 27°C with a 12L:12D photoperiod (Paul Eady, University of Lincoln, personal communication).

### Life History Responses

#### Study Design

**Fig. 1.**
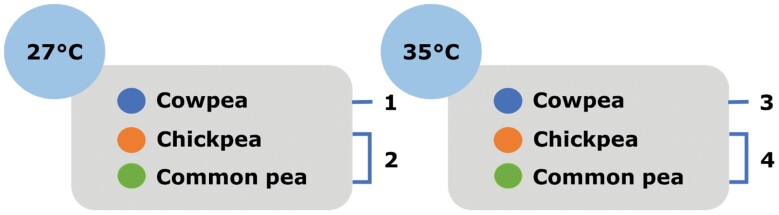
Experimental design for examining the transcriptomic and life history responses to: (1) control treatment (cowpea and 27°C as the ancestral bean and ambient temperature), (2) resource change (i.e., chickpea or common pea as a novel resource at the ancestral temperature of 27°C), (3) temperature change (i.e., cowpea as the ancestral bean at a novel temperature of 35°C) and (4) combined resource and temperature change (i.e., chickpea or common pea as a novel resource combined with a novel temperature of 35°C). Numbers in the diagram refer to these 4 types of imposed environmental change.

To determine life history responses to single and multidimensional changes in temperature and resource components of the environment, *C. maculatus* beetles were exposed to 1 of 3 treatments: (i) control (ancestral resource of cowpea and ancestral temperature of 27°C), (ii) novel resource at the ancestral temperature, (iii) novel temperature but ancestral resource, or (iv) novel resource and novel temperature (Fig. 1). For the novel temperature, we selected 35°C. Pilot studies testing elevated temperatures of 32°C, 35°C, and 37°C revealed 35°C as near but not surpassing the upper limit of the thermal reaction norm (Unpublished Data). For the novel resource, chickpea (*Cicer arietinum*) or common pea (*Pisum sativum*) was provided. Pilot studies further found that total offspring produced by a population introduced onto common pea was 49 ± 0.06 (SE) adult offspring produced per female after 2 generations, compared with 67.9 ± 2.83 (SE) offspring per female on cowpeas (Leonard and Lancaster 2022). Chickpea was also selected as a novel resource as it caused a 27.6 ± 0.02% decrease in offspring produced in previous studies (Price et al. 2017). All rearing was performed in a programmable incubator with a 12L:12D photoperiod (LMS model 280NP refrigerated incubator; Kent, UK). Beetles were reared in these treatments for 2 generations to ensure resource and temperature shifts were experienced by all life stages.

Each treatment began with 40 F_0_ replicate pairs (each pair comprising of 1 male and 1 female) generated from beetles selected randomly from the stock culture (cowpea at 27°C) and placed in a petri dish with 10g of beans (cowpea, chickpea or common pea; *n* = 40 F_0_ pairs per resource type) on which to oviposit at 27°C or 35°C (*n* = 240 total pairs). Upon adult emergence of F_1_ generation, 2 beetles, 1 virgin male, and 1 virgin female, were taken from each successfully emerging replicate per treatment. Within each treatment, each beetle was then paired with another beetle of the opposite sex from a different replicate to prevent pairing of siblings. After loss of some families that failed to successfully breed, this created *n* = 184 total F_1_ mated pairs (see Table 1 for total number of F_1_ pairs per treatment). These pairs were each established on 10 g of fresh beans of the same bean type and cultured in the same temperature as their parents had experienced. Except for 2nd generation larvae sacrificed for molecular analysis before reaching adulthood (see below), each generation experienced an average of 23 days in these conditions, the duration dependent on the condition. Data collection on beetle performance included F_1_ fecundity (total number of eggs), F_2_ developmental period (number of days from egg to adult) and F_2_ offspring viability (total number of F_2_ emerged adults divided by total number of eggs laid by F_1_).

**Table 1.** F_1_ sample size per treatment for life history analysis

Temperature	Cowpea (*n*)	Chickpea (*n*)	Common pea (*n*)
27°C	31	23	27
35°C	35	35	33

#### Statistical Analysis

All statistical analyses for life history responses were performed in R version 4.1.1 using the ‘stats’ package and figures were plotted using the ‘ggplot2’ package (Wickham 2016, R Core Team 2021). The generalized linear models were applied to the test for host resource and temperature as main effects of an interaction. The separate models were fit for each trait measured. F_1_ fecundity was modeled fitting a general linear model with a quasipoisson error distribution, as this represented count data with large dispersion parameters (13.77). F_2_ developmental period (number of days from egg to adult) was fitted using a gamma distribution (dispersion parameter = 0.008), and F_2_ offspring viability was fit with a quasibinomial distribution (dispersion parameter = 4.94), modeling the number of offspring emerging weighted by the total fecundity. In the latter case, fecundity was also added as a covariate to further account for competition effects that reduce larval survival in larger clutches. Combined effects of resource and temperature were assessed with cowpea and 27°C controls as the baseline and with temperature and resource fitted as categorical variables. For means, standard deviation and standard errors of each life history within each treatment, see Supplementary Table S1.

### RNA Sequencing

#### Study Design

To examine transcriptomic responses to single and multidimensional environmental change in *C. maculatus*, 4 whole F_2_ larvae individuals, each from different families, were collected from each of the following treatments: cowpea-27°C (control treatment), chickpea-27°C (resource change), cowpea-35°C (temperature change), and chickpea-35°C (multidimensional resource and temperature change), resulting in 16 samples in total. The larval stage was chosen as it is the only actively feeding life stage (Wasserman and Futuyma 1981); therefore, we believed this stage would be the most relevant for assessing gene expression responses to resource and temperature change. Common pea did not produce a sufficient number of viable larval individuals for sequencing; therefore, for the treatments involving resource change, larvae were sequenced from chickpea only. F_2_ larvae of the 4th instar, estimated by their large size and high activity rate compared to the earlier instars (Rêgo et al. 2020), were extracted by gently cracking the bean open with a mortar and pestle. Larvae individuals were immediately frozen at −70°C prior to total RNA extraction.

#### Sample Preparation

Individual larvae samples were homogenized mechanically, and total RNA was extracted using a standard Trizol-chloroform procedure (Thermo Fisher Scientific, Lynch et al. 2016). RNA pellets were then reconstituted in 30 µl RNase-free DNase water (Invitrogen, Thermo Fisher Scientific) and RNA concentration and quality were determined using Qubit fluorometry (Thermo Fisher Scientific).

#### Library Preparation and Sequencing

Messenger RNA (mRNA) library preparation and sequencing was performed by Novogene Co. Ltd. (Cambridge, UK). Specifically, mRNA was purified from total RNA using poly-T oligo-attached magnetic beads. After fragmentation and cDNA synthesis, adapters were ligated onto the strands which were then amplified and purified. Quantified libraries were sequenced with the NovaSeq 6000 PE150 platform (Illumina) to produce 20 million 150-bp paired-end reads. These raw sequence data have been submitted to the National Center for Biotechnology Information (NCBI) database under the BioProject number PRJNA1026102.

#### Quality Control, Genome Mapping, and Transcript Annotation

Raw reads were filtered through fastp to remove adapter reads, poly-N sequences and low-quality reads (Chen et al. 2018b). Read quality of clean reads was also scored using Q20 and Q30, with sequencing error rates calculated as 1 in 100 and 1000, respectively. Only bases with a quality of greater than Q15 qualified to pass filtering, reads with the number of N bases >15 were discarded, and the percentage of mismatched bases to detect overlapped regions of paired-end reads was set at maximum 10%, with the remaining parameters set as default. HISAT2 software (Kim et al. 2019) was then used to analyze each paired-end clean read and align the clean reads to the *C. maculatus* reference genome (NCBI taxon ID: 64391; GenBank: GCA_900659725.1; genome assembly number: ASM90065972v1), using a Hierarchal Graph Full-text in Minute space (HGFM) index. Gene-level transcript assemblies were then identified and assigned PFAM database annotations (Finn et al. 2008) in Stringtie (Pertea et al. 2015), using the reference genome annotation file (Sayadi et al. 2019). FeatureCounts command-line was then used to quantify read counts per gene (Liao et al. 2013). All library preparation, sequencing, assembly, mapping and annotation steps were performed by Novogene Co. Ltd. (Cambridge, UK) using proprietary pipelines.

#### Differential Expression Analysis Using DESeq2

DESeq2 analyses in R (Anders and Huber 2010) were performed to identify differentially expressed genes in each treatment via pairwise comparisons, using generalized linear model equations created by Love et al. (2014). Although pairwise comparisons were performed with all treatments, only 3 pairwise comparisons have been reported here (resource change: chickpea-27°C vs. cowpea-27°C; temperature change: cowpea-35°C vs. cowpea-27°C; resource and temperature change: chickpea-35°C vs. cowpea-27°C). The *P* values were adjusted for multiple comparisons using the Benjamini and Hochberg’s approach for controlling the False Discovery Rate (FDR). Expression ratios of treated samples relative to control treatment with a log2 fold change of >0 (upregulated), <0 (downregulated) and an adjusted *P* value of <0.05 were considered as differently expressed. We tested whether the changes in gene expression were correlated in each pair of treatments, using Spearman’s rank correlation on the log 2-fold change values of each shared gene. Using the fragments per kilobase per million mapped reads (FPKM) values of the differentially expressed genes, heat maps were used to visualize hierarchical clustering of genes with similar or different expression patterns across treatments (created by Novogene Co. Ltd. using the ‘ComplexHeatmap’ R package in BiocManager [Morgan 2022]).

To identify genome-wide differences in expression among treatments, a between-group analysis (using the ‘made4’ R package, Culhane et al. 2002, 2005) was conducted to visualize divergence of each thermal treatment group within and among populations, for all transcripts. In our between-group analysis, we ordinated groups of samples according to the principal component analysis (PCA) and then projected individual samples back onto the first 2 PCA axes.

#### Differential Expression Analysis Using edgeR

To specifically test for significant multiplicative effects of resource and temperature shifts on gene expression, the transcript read counts from FeatureCounts were used for analysis in the ‘edgeR Bioconductor’ package (Robinson et al. 2010). Specifically, read counts were normalized using the trimmed mean of *M*-values normalization method (TMM; Robinson and Oshlack 2010). We used multidimensional scaling plots to check for consistency of samples within each category and then conducted genewise general linear models (GLMs) in edgeR to estimate the genewise dispersion estimates over all genes and identify transcripts with expression patterns that were better explained across all samples by temperature + host resource + temperature × host resource (i.e., nonadditive effects) than by additive effects of temperature + host resource alone, where significance of the interaction effect was established using likelihood ratio tests for each transcript. In this analysis, *P* values were adjusted for multiple comparisons using the Benjamini and Hochberg methods and with an FDR cut-off of 0.05 (Robinson et al. 2010), but we did not specify any particular minimum threshold for magnitude of effect. Hypergeometric tests were used to test whether the PFAM annotations were over-represented in these transcripts due to being more common among all transcripts that were tested. Finally, to examine the patterns in gene functionality, each of these transcripts was given a summarized function based on the gene descriptions from PFAM annotation. To facilitate interpretation of expression trends, transcripts were grouped into 1 of 50 specific functions, and functions were then plotted together based on the following common patterns of expression across the treatments, determined by visual interpretation (Fig. 4): (i) upregulation in the multi-dimensional treatment only; (ii) upregulation in both the control and in the multi-dimensional treatments, but not single dimensional treatments; (iii) downregulation in both control and multi-dimensional treatments compared to single dimensional treatments; (iv) upregulation in the temperature treatment only; and (v) downregulation in the temperature treatment only.

## Results

### Life History Responses

#### Fecundity

**Fig. 2.**
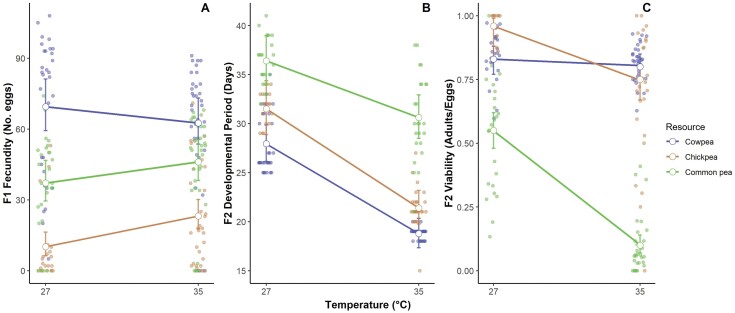
Combined effects of host resource and temperature on A) fecundity (*n* = 182), B) developmental period (*n* = 154) and C) offspring viability (*n* = 144). Error bars indicate the predicted values with the 95% confidence intervals around the fitted general linear models.

The generalized linear models revealed that there was no significant main effect of temperature on fecundity (*P* = 0.36). However, there was an effect of host resource. When beetles were reared on chickpea or common pea, they had significantly reduced fecundity compared to beetles emerging from cowpea (effect of chickpea: *P* = 2.71e−12; effect of common pea: *P* = 1.77e−05). In addition, there was a significant chickpea × temperature interaction effect on fecundity, where individuals reared on chickpea, but not other host resources, showed increased fecundity at 35°C compared to 27°C (*P* = 0.002; Table 2, Fig. 2A).

**Table 2.** The generalized linear models explain the combined effects of host resource and temperature on fecundity, offspring viability (with fecundity as a covariate), and developmental period (baseline = cowpea, 27°C). Significant relationships are indicated in bold

	Fecundity (*n* = 182)				Developmental period (*n* = 154)				Offspring viability (*n* = 144)			
Temperature-treatment	Est	SE	*t*	*Pr*(*>*|*t*|)	Est	SE	*t*	*Pr*(*>*|*t*|)	Est	SE	*t*	*Pr*(*>*|*t*|)
Intercept	4.24	0.08	53.01	<2e−16	0.04	0.0006	61.33	<2e−16	1.39	0.41	3.37	0.001
35°C	−0.10	0.11	−0.92	0.36	0.02	0.001	16.86	**<2e**−**16**	−0.17	0.20	−0.85	0.40
Chickpea	−1.92	0.26	−7.52	**2.71e**−**12**	−0.004	0.0009	−4.37	**2.34e**−**05**	1.57	0.80	1.96	0.05
Common pea	−0.63	0.14	−4.41	**1.77e**−**05**	−0.008	0.0008	−10.64	**<2e**−**16**	−1.39	0.26	−5.27	**5.14e**−**07**
Fecundity	NA	NA	NA	NA	NA	NA	NA	NA	0.004	0.005	0.77	0.44
Chickpea × 35°C	0.93	0.30	3.10	**0.002**	0.002	0.001	−1.64	0.10	−1.90	0.81	−2.35	**0.02**
Common pea × 35°C	0.32	0.19	1.70	0.09	−0.01	0.001	−9.40	**<2e**−**16**	−2.21	0.31	−7.02	**9.43e**−**11**

#### Developmental Period

An increase in temperature had an overall significant effect of reducing developmental period (*P* = < 2e−16). Additionally, both novel host resources significantly increased developmental period compared to cowpea (effect of chickpea: *P* = 2.34e−05; effect of common pea: *P* = < 2e−16). A significant host resource × temperature interaction effect on developmental period confirmed that beetles reared on common pea exhibited a less steep reduction in developmental period at 35°C versus 27°C than beetles reared on control beans (*P* = < 2e−16; Table 2, Fig. 2B).

#### Offspring Viability

Elevated temperatures did not significantly reduce offspring viability (*P* = 0.40). While chickpea had no significant overall effect on offspring viability compared to cowpea (*P* = 0.05), common pea significantly reduced offspring viability compared to cowpea (*P* = 5.14e-07). The generalized linear models further confirmed significant host resource × temperature interaction effects on offspring viability, with individuals reared on chickpea and common pea showing a stronger decrease in offspring viability at 35°C versus 27°C, in contrast to controls (*P* = 0.02 and 9.43e−11, respectively). Finally, fecundity did not significantly affect viability of offspring when added as a covariate (*P* = 0.44; Table 2, Fig. 2C).

### RNA Sequencing

**Fig. 3.**
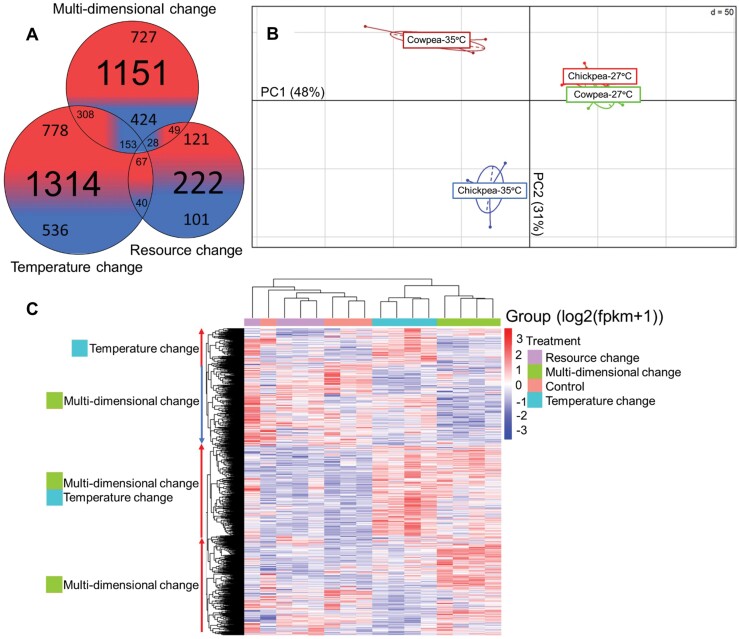
Variation in gene expression under temperature change (cowpea-35°C), resource change (chickpea-27°C) and multidimensional change (chickpea-35°C) in comparison to cowpea-27°C control. A) Venn diagram of significantly differentially expressed genes within treatments and shared between treatments. Size of circles correspond to the number of differentially expressed genes in each treatment, the smaller numbers and the associating colours within each circle represent the number of upregulated and downregulated genes within each treatment, and overlapping circles represent the number of shared up and downregulated genes between treatments. B) A between-group PCA of all 21,259 expressed transcripts in each treatment. Points represent individuals and are connected by solid lines. The major and minor axes of each confidence ellipse are represented by dashed lines. C) Hierarchical clustering heat map with all differentially expressed genes in all larval samples showing similar expression patterns across the 4 treatments. Along the left column, arrows and their color indicate direction of differential expression of genes showing prominent hierarchical clustering within certain treatments, where “up” arrows indicate upregulated genes clustered in a treatment in comparison to the control treatment and “down” arrows indicate downregulated genes clustered in a treatment in comparison to the control treatment.

In total, 21,259 transcripts were generated using NovaSeq 6000 PE150 Illumina platform (for a summary of mapping statistics, see Supplementary Table S2). Of the 21,259 transcripts used in DESeq2 for differential expression analysis across temperature and resource treatments, 1314 transcripts were differentially expressed in the temperature treatment (cowpea-35°C) (Fig. 3A). Of these, 778 (60%) were upregulated and 536 (40%) were downregulated in comparison to the control treatment (cowpea-27°C). Only 222 transcripts were differentially expressed in the resource treatment (chickpea-27°C), where 121 (54%) transcripts were upregulated and 101 (45%) transcripts were downregulated in comparison to the control treatment. Within the multidimensional treatment (chickpea-35°C), 1151 transcripts were differentially expressed, with 727 (63%) of the transcripts upregulated and 424 (37%) downregulated in comparison to the control. Overall, more transcripts were differentially expressed under temperature change or combined resource and temperature change than in resource change alone.

Despite strong positive correlations observed between the log2 fold change values shared across treatments (temperature vs. resource change: Spearman’s *ρ* = 0.96, *P* < 2.2e−16; temperature vs. multidimensional change: Spearman’s *ρ* = 0.92, *P* < 2.2e−16; resource vs. multidimensional change: Spearman’s *ρ* = 0.88, *P* = < 2.2e−16; Supplementary Fig. S1), there was little overlap in the number of shared differentially expressed genes between treatments: 67 genes that were upregulated in the temperature treatment were also upregulated in the resource treatment, while 40 genes shared downregulation and 1 gene showing the opposite pattern (i.e., upregulated in 1 treatment while downregulated in the other treatment). Moreover, only 308 out of 778 genes that were upregulated in temperature change were also upregulated in the multidimensional treatment, and 153 out of 536 genes shared downregulation. Similarly, only 49 out of 121 genes upregulated in resource change were also upregulated in the multidimensional treatment, with 28 out of 101 genes sharing downregulation and 5 genes showing the opposite pattern (Fig. 3A). This indicates that a largely different set of genes were differentially expressed under multidimensional resource and temperature change compared to under single dimensional changes in resource and temperature. For information on the differential analysis results of all genes from each treatment pairwise comparison, and total number of shared differentially expressed transcripts between each pairwise comparison, see Supplementary Tables S3 and S4.

Between-group analysis of all 21,259 transcripts according to the PCA revealed that the majority of the variation among groups was explained by temperature treatment (PC1 = 48% of variation; Fig. 3B). Among-resource variation in gene expression (PC2 = 31% of variation) was lower for individuals from ambient temperatures (i.e., cowpea-27°C and chickpea-27°C; Fig. 3B, right side) than for individuals that experienced multidimensional change (i.e., cowpea-35°C and chickpea-35°C; Fig. 3B, left side).

Heat map clustering analysis show that chickpea-27°C and cowpea-27°C treatments cluster together (Fig. 3C, left 2 columns), indicating that larvae from the resource treatment shared similar differential expression patterns to the control treatment. Conversely, cowpea-35°C and chickpea-35°C treatments cluster together (Fig. 3C, right 2 columns), indicating that larvae from the temperature treatment shared similar differential expression patterns to larvae from the multidimensional resource and temperature treatment. Cowpea-35°C and chickpea-35°C treatments cluster away from cowpea-27°C and chickpea-27°C treatments, resulting in differences in differential gene expression patterns under temperature change and multidimensional resource and temperature change compared to under control conditions (Fig. 3C, arrow annotations).

**Fig. 4.**
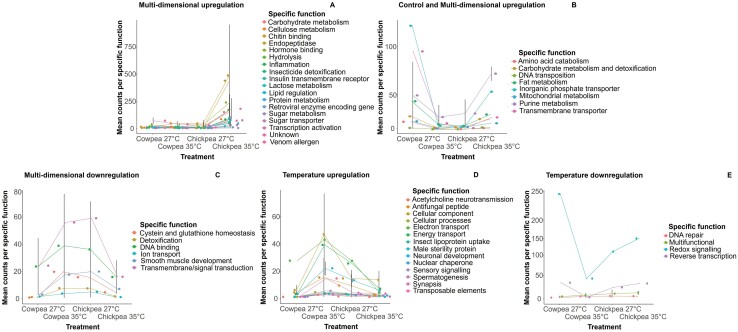
Genes significantly differentially expressed according to type of host resource × temperature interaction: A) genes with 18 different specific functions showing overall trends of upregulation in expression in response to multidimensional resource and temperature change (n = 69). B) Genes with 8 different specific function that were overexpressed in both the control and in host resource × temperature change (*n* = 9). C) Genes with 6 different specific functions that show the reverse trend to B) and are under expressed in both control and temperature × host resource (*n* = 7). D) Genes with 14 different specific functions that are overexpressed under temperature change (*n* = 23). E) Genes with 4 different specific functions that show the reverse trend to D) and are under expressed with temperature change (*n* = 7). Error bars indicate the standard error of the mean when multiple genes fall under the same specific function.

Of the 21,259 transcripts analyzed in edgeR for significant interactions between temperature and resource environment effects on gene expression, 115 transcripts were significantly over- or under-expressed in the chickpea-35°C multidimensional treatment compared to what would be expected under an additive model. Hypergeometric tests showed that the PFAM annotation of 110 of these transcripts were not over-represented among the 115 transcripts as they are not common among the 21,259 transcripts tested (Supplementary Table S5). 64 of those transcripts (56%) were upregulated (compared to the cowpea-27°C control treatment), while 51 transcripts (44%) were downregulated. No transcripts exhibited a significant interactive effect on expression consistent with up or down regulation in the resource treatment only. Transcripts showing an overall pattern of upregulation in the multidimensional treatment were mapped to genes relating to energy and toxin metabolism, growth and development, immune function, macromolecular transport and exoskeleton structure, with some genes of unknown function (functional trend A, Fig. 4A). Genes involved in other forms of transport and energy and toxin metabolism also showed upregulation under control and multidimensional treatments (functional trend B, Fig. 4B). Conversely, transcripts showing an overall downregulation pattern in the multidimensional and control treatments were mapped to genes involved in signaling and other forms of toxin metabolism and growth and development (functional trend C, Fig. 4C). Finally, transcripts that were upregulated only in the temperature treatment are involved in cellular structure, reproduction, neurotransmission and other forms of immune and transport functions (functional trend D, Fig. 4D) and genes involved in DNA repair, signaling and reverse transcription showed opposite trends of downregulation in the temperature treatment (functional trend E, Fig. 4E). For more details on individual genes and function, see Supplementary Table S5.

## Discussion

We integrated transcriptomic and phenotypic assays to examine insect responses to single and multidimensional environmental change. We determined that temperature and resource change independently and interactively influenced fecundity, developmental period and offspring viability in *C. maculatus* in a context-dependent manner (Fig. 2). As a single dimensional change, resource change had a negative effect on each life history trait measured by reducing fecundity, increasing developmental period and reducing offspring viability. Temperature had no overall effect on fecundity and offspring viability, although increasing temperature had an overall effect of reducing developmental period. Moreover, the combination of resource and temperature multidimensional change had a significant interaction effect on life history traits measured, resulting in greater changes in both fecundity and offspring viability than in either temperature or resource change alone (but not developmental period for chickpea-35°C).

We show that many shared genes are co-regulated in response to either temperature or resource shifts, suggesting candidate pleiotropic responses, which can further potentially enable new adaptations to other aspects of the environment following periods of change (e.g., Leonard and Lancaster 2022). However, in contrast to the effect of single dimensional change on life history traits, which were greater for resources than for temperature, temperature was a more important factor driving changes in gene expression patterns in *C. maculatus* (Fig. 3A–C). This suggests that the shifts in gene expression confer a plastic response to increased temperature without observing a negative effect on life history. The upregulation of many metabolic transcripts (see below) may explain the reduction in developmental period observed with increasing temperatures on all resources. Temperature is known to increase the metabolic rate in insects (Deutsch et al. 2018). In addition, a shorter developmental period is beneficial for insect pests by reducing generation times, as well as shortening the time of exposure to heat in the vulnerable larval stages (Bale et al. 2002, Brommer et al. 2002, Menéndez 2007, Massot et al. 2021). Therefore, increased temperature may well have induced changes in metabolic gene expression resulting in a decrease in developmental period. Conversely, resource change caused the least differential expression compared to the controls (Fig. 3A) despite showing the largest differences in life history traits measured (Fig. 2). The relative lack of change in gene expression associated with life history changes on a novel host could reflect differences in how insects respond to temperature vs. resources overall (i.e., more variation in genes associated with temperature responses than there are for resource responses). In addition, a decrease in fecundity and developmental period when shifted to a novel resource, but no change in response to temperature, may be due to the high host specialization in the beetles towards cowpea, limiting their ability to cope with novel nutritional changes (Kelley and Farrell 1998) and implying that they lack the adaptive capacity to shift to a novel resource via changes in gene expression.

### Nonlinear Transcriptomic Responses to Multidimensional Environmental Change

Multidimensional change in resource and temperature resulted in different sets/patterns of differentially expressed genes than expected under an additive model (Fig. 3A and C), with 115 genes responding significantly nonadditively to multidimensional change (Fig. 4). Moreover, there was low overlap between genes differentially expressed in pairwise comparisons between multi- and single dimensional treatments. These lines of evidence support the hypothesis that multidimensional changes interactively act upon gene expression. Among the genes exhibiting significant resource × temperature interaction effects, the majority of these were found only to respond under multidimensional changes, with 69 out of 115 genes showing overall upregulation at chickpea-35°C (Fig. 4A), which may reflect a difference in physiological requirements under multidimensional change. Differential expression of these key genes may therefore be required to reach trait optimum under the multidimensional environmental conditions and minimize trade-offs between traits (Kaunisto et al. 2016, Orr et al. 2020). The expression of multiple genes was similar under multidimensional change and at cowpea-27°C, but were up or downregulated in single dimensional changes (Fig. 4B and 4C). Similarly, while the remaining nonadditive differentially expressed genes were overall up or downregulated in response to temperature change alone, the expression of these genes under multidimensional change are similar to expression levels under baseline control conditions (Fig. 4D and E). This implies that expression of certain functional genes returns to baseline expression levels under multidimensional change, which may indicate loss of ability to express beneficial plasticity under more complex environments (McEwen 1998).

### Upregulated Transcripts Under Resource × Temperature Change

We found several protective mechanisms upregulated nonadditively under multidimensional environmental change (Fig. 4A), indicating that co-expression of multiple response mechanisms may allow *C. maculatus* to cope with simultaneous changes to the environment. Transcripts associated with chitin binding were the most upregulated transcripts in the multidimensional treatment. Chitin is an important scaffolding protein found in the exoskeleton, epidermis and gut epithelium in insects (Merzendorfer and Zimoch 2003). Previous studies have also found upregulation of chitin in whiteflies (*Bemisia tabaci*) adapted to a less optimal plant source, which indicates an important role of upregulating insect cuticle proteins to combat heat desiccation and plant defenses (Tadmor et al. 2022). The second most upregulated transcript in response to multidimensional change was the endopeptidase, cathepsin L (Supplementary Table S5). Expression of cathepsin L is gut-specific in many insects including aphids and stink bugs (Cristofoletti et al. 2003, Bansal and Michel 2018) and is an important lysosome component for protein degradation (Shu et al. 2019). Upregulation of cathepsin L was found in thermally stressed Asian citrus psyllids (*Diaphorina citri*) (Shu et al. 2020) and brown marmorated stink bugs (*Halyomorpha halys*) fed a generalist diet (Bansal and Michel 2018), suggesting its importance in responses to both heat and resource changes. Upregulation of transcripts associated with insecticide detoxification (specifically: cytochrome P450, esterase B1 (carboxylesterase) and ABC-2 family transporter protein, Supplementary Table S5) during multidimensional changes is consistent with studies showing a correlation between upregulation of these detoxifying genes and higher tolerance to subsequent insecticide exposure in insects by breaking down xenobiotic compounds (Chen et al. 2018a, Amezian et al. 2021, Lu et al. 2021). Further, upregulation of detoxification genes like cytochrome P450s in response to high temperatures in other insect studies (Liu et al. 2017, Wang et al. 2021) suggests adaptive cross tolerance can occur in insects exposed to insecticides and temperature change. In addition, upregulation of the sugar transporter for trehalose (deemed the insect ‘blood’ sugar; Thompson 2003, Supplementary Table S5) at chickpea-35°C supports other study findings that trehalose upregulation and trehalose accumulation increased heat resistance in the Antarctic midge fly (*Chymomyza costata*) by stabilizing heat-sensitive proteins during temperature change (Benoit et al. 2009, Gotcha et al. 2018). Trehalose accumulation was also found to contribute to high freeze tolerance in drought-induced malt flies (*C. costata*) by forming a cryoprotectant mixture around the tissues (Hůla et al. 2022). Furthermore, upregulation of transcripts involved in sugar transportation and carbohydrate, lactose and sugar metabolism during multidimensional change may result in increased levels of circulating sugars in the beetles—which has previously been found to occur in thermally stressed insects to provide a quick energy source as well as providing materials for macromolecular stabilization and reactive oxygen species scavenging to reduce cellular damage (Teets et al. 2012, Sulmon et al. 2015, Bueno et al. 2023). Importantly, our study provides novel insight into how these characterized mechanisms, commonly found in other insect studies to respond only to a single dimensional change, or to an additional stressor after prior exposure to a different stressor (Benoit et al. 2009, Gotcha et al. 2018), respond multiplicatively to simultaneous multidimensional environmental change in *C. maculatus* and potentially in other insects.

### Differentially Expressed Transcripts Under Single Dimensional Change

We found nonadditive antagonistic effects of temperature change and resource change alone on a number of mechanisms, where transcriptional activity was greater under single dimensional changes than under multidimensional change (Fig. 4C–E). For instance, transcripts associated with the cytoskeleton were upregulated in the temperature treatment (cellular component line, Fig. 4D). Previous studies have shown that cytoskeletal rearrangement occurs during cold damage repair in the flesh fly (*Sarcophaga bullata*) and in the northern house mosquito (*Culex pipiens*) (Kim et al. 2006, Teets et al. 2012), indicating the importance of transcriptional plasticity of cytoskeletal genes in insects under temperature change. Moreover, there appears to be variation in transcriptional activity of genes involved in DNA damage repair and apoptosis, processes of which are known to be induced during stress (Kültz 2005), within the temperature and resource treatments. Ubiquitin-protein ligase, involved in many cellular processes including apoptosis and DNA repair (Wu et al. 2022, Supplementary Table S5), was upregulated in the temperature treatment (cellular processes line, Fig. 4D), and gamma-glutamyltranspeptidase 1, which aids in cysteine and glutathione homeostasis during oxidative stress (Zhang et al. 2005), was upregulated in response to temperature and resource change (Fig. 4C). Meanwhile, serine/threonine-protein kinase (Ataxia telangiectasia and Ras3-related protein [ATR]), involved in DNA repair by activating cell cycle arrest upon DNA damage in eukaryotes (Sancar et al. 2004), and thioredoxin, involved in redox signaling (Kanzok et al. 2001), were downregulated during temperature change (Fig. 4E). This variation in expression of transcripts involved in DNA repair and cell death could be because mechanisms involved in apoptosis may be cell-specific in responding to a certain environmental change, thus environmental tolerance may be associated with activation or inhibition of these mechanisms depending on the cells targeted (Sulmon et al. 2015). Together, these mechanisms associated with prevention of structural and internal damage, while acting nonlinearly, are less responsive to combined changes in temperature and resource.

### Transcriptomic Versus Phenotypic Responses

When considering the effects of single dimensional changes on the life history and transcriptome responses in *C. maculatus*, there appear to be opposite trends for levels of overall change induced in the transcriptome vs. the phenotype: while changes in resource had more of an effect on life history than temperature (by reducing fecundity, increasing developmental period and reducing offspring viability) temperature had the greatest transcriptional effects (Fig. 2 and 3). The greater increase in differential expression under temperature change compared to resources may be expected if the changing gene expression causes phenotypic buffering against detrimental impacts of environmental change (Swaegers et al. 2020), as observed with little overall effect of elevated temperatures on life history responses (Fig. 2). Similarly, the combined environment provided different environmental cues and thus resulted in different physiological requirements (under which multiple genes that exhibited putatively adaptive changes in expression under temperature or resources alone were not similarly differentially expressed under resources and temperature in combination), so plasticity in life history traits acted differently. This effect (of loss of gene expression plasticity under more complex environmental change) was also observed in damselflies (*Ischnura elegans*), where larvae from northern latitudes (that naturally experience lower temperatures than damselflies from southern latitudes) showed plastic responses in gene expression but no response in growth or development rate under mild warming compared to larvae from southern latitudes that showed the opposite phenotypic and transcriptomic responses (Van Dievel et al. 2019, Swaegers et al. 2020). This suggests that genetic compensation may drive plastic responses during future climate warming in ectotherms.

## Conclusion

Our results provide valuable information for understanding the role of transcriptional and phenotypic responses to multidimensional environmental change in insects. We conclude that while single dimensional changes are useful for understanding the mechanisms underpinning species responses to environmental change, they cannot determine life history and transcriptomic responses to multidimensional environmental change exhibited in our current climate. Future studies should assess insect responses to multidimensional environmental change to better understand how species are responding to change in a heterogeneous environment, particularly using experimental evolution over multiple generations, as some patterns may only be apparent in longer term. Such studies may help contribute to theory on predicting pest outbreaks under future climate scenarios.

## Supplementary Material

ieae106_suppl_Supplementary_Tables_S1-S5

ieae106_suppl_Supplementary_Figures_S1
