# Supplementary figures and images for: Nonlinear transcriptomic responses to compounded environmental changes across temperature and resources in a pest beetle, Callosobruchus maculatus (Coleoptera: Chrysomelidae)
Source: J Insect Sci. 2024 Dec 13;24(6):11. doi: 10.1093/jisesa/ieae106 (PMC11638975; doi:10.1093/jisesa/ieae106)

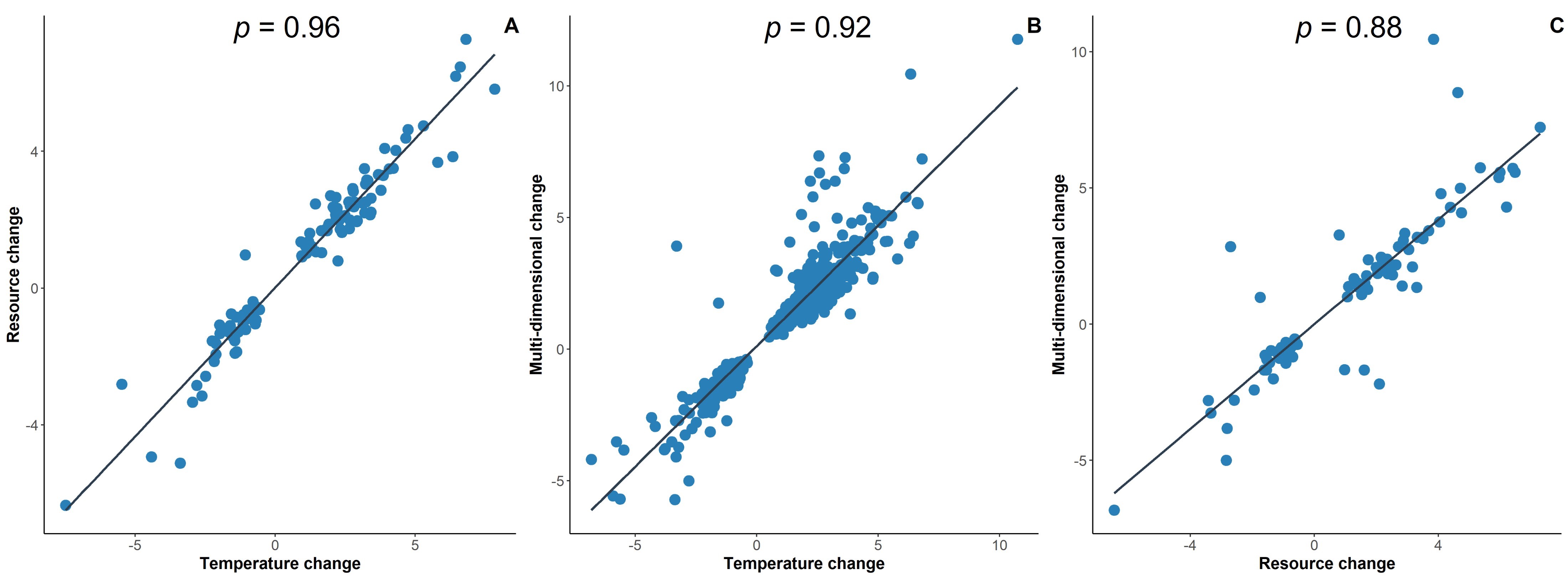

Supplement: ieae106_suppl_Supplementary_Figures_S1 [file ieae106_suppl_supplementary_figures_s1.jpeg]
